# Effect of multidisciplinary team care on patient survival in chronic hepatitis B or C hepatocellular carcinoma

**DOI:** 10.3389/fonc.2023.1251571

**Published:** 2023-12-21

**Authors:** Yu-Chen Tseng, Pei-Tseng Kung, Cheng-Yuan Peng, Wen-Yu Chou, Wen-Chen Tsai

**Affiliations:** ^1^ Department of Public Health, China Medical University, Taichung, Taiwan; ^2^ Department of Health Services Administration, China Medical University, Taichung, Taiwan; ^3^ Division of Gastroenterology and Hepatology, Department of Internal Medicine, Taichung Armed Forces General Hospital, Taichung, Taiwan; ^4^ Division of Gastroenterology and Hepatology, Department of Internal Medicine, Tri-Service General Hospital, National Defense Medical Center, Taipei, Taiwan; ^5^ Department of Healthcare Administration, Asia University, Taichung, Taiwan; ^6^ Department of Medical Research, China Medical University Hospital, Taichung, Taiwan; ^7^ Center for Digestive Medicine, Department of Internal Medicine, China Medical University Hospital, Taichung, Taiwan; ^8^ School of Medicine, China Medical University, Taichung, Taiwan

**Keywords:** hepatocellular carcinoma, multidisciplinary team, hepatitis B virus, hepatitis C virus, survival

## Abstract

**Introduction:**

Multidisciplinary team care coordinates with medical teams to improve the quality of cancer care. This study explored multidisciplinary team care in hepatitis B or hepatitis C virus-related hepatocellular carcinoma patients from the time of diagnosis to the first-time treatment interval and investigated treatment outcomes and prognosis.

**Methods:**

This retrospective cohort study included data from a nationwide population from 2007 to 2016. Data were collected from the Taiwan Cancer Registry Database, linked to the Taiwan National Health Insurance Research Database. Propensity score matching was applied at a ratio of 1:2 to reduce the selection bias. A multiple regression model with generalized estimating equations was used to analyze whether multidisciplinary team care affected the diagnosis-to-treatment interval. The stratified Cox proportional hazards model examined whether involvement in multidisciplinary team care influenced survival status.

**Results:**

A total of 10,928 and 21,856 patients with hepatocellular carcinoma received multidisciplinary and non-multidisciplinary care, respectively. Participants with multidisciplinary care had a longer diagnosis-to-treatment interval but a lower risk of cumulative cancer death (HR=0.88, 95% CI:0.84-0.92). In patients with intermediate- to advanced-stage hepatocellular carcinoma, multidisciplinary team care has obvious benefits for improving survival.

**Conclusion:**

Patients with hepatocellular carcinoma who participated in multidisciplinary team care had a longer diagnosis-to-treatment interval but a lower risk of cancer death. Patients with intermediate- to advanced-stage hepatocellular carcinoma who received multidisciplinary team care significantly benefited from this outcome. Hospitals should provide HCC patients with multidisciplinary team care to improve cancer care.

## 1 Introduction

Primary liver cancer, also known as hepatocellular carcinoma (HCC), has high incidence and mortality rates. According to the latest report from the International Agency for Research on Cancer (IARC), HCC is estimated to be the sixth leading cause of new cancer cases and the second leading cause of cancer-related deaths worldwide in 2020 (1). The latest Taiwan Cancer Registry Annual Report stated that HCC was the fifth leading cause of cancer (11,272 people per 100,000 person-years) and the second leading cause of cancer-related deaths (7,881 people per 100,000 person-years) in Taiwan in 2019 (2). The major risk factor for HCC is the gradual progression of chronic hepatitis to liver fibrosis and cirrhosis following hepatitis B virus (HBV) or hepatitis C virus (HCV) infection (3). About 80% of HCC cases are associated with chronic hepatitis B (CHB) or chronic hepatitis C infections (3). Other common risk factors for HCC include alcoholic hepatitis, non-alcoholic fatty liver disease, obesity, and diabetes (4). With liver injury or inflammation, liver cells respond to simultaneous regeneration and fibrosis. Liver fibrosis also regulates inflammatory cell activity in the liver. More than 80% of the patients diagnosed with HCC have cirrhosis (5). Liver dysfunction and worse tumor burden (multiple nodules or large tumors) are associated with a poorer prognosis for patients with HCC (6). Liver disease is usually insidious in onset, with inconspicuous symptoms; therefore, HCC has previously been diagnosed in the middle to late stages (7). Since HCC is always diagnosed at a non-early stage, treatment options are severely limited (6). There are no specific specialists who are sufficiently trained to meet the needs of this patient population (6).

Multidisciplinary team (MDT) care originated in a cancer care study that recommended integrated medical staff in each category and across specialists to provide consistent, high-quality individualized care (8). The purpose of MDT care is to help healthcare professionals make better medical decisions, improve patient outcomes, and optimize the quality of the healthcare system (9). MDT care is widely used for the clinical management of various cancers and chronic diseases (10–16). Some studies have reported that MDT care can extend the survival of late-stage non-small cell lung cancer and oral cavity cancer patients (17, 18), lower the mortality risk of colorectal, oral cavity, and esophageal cancer patients (19–21), decrease the frequency of emergency department visits of lung and colorectal cancer patients (22, 23), reduce the 14-day readmission rates of colorectal cancer patients (24), and lowering the relative risk of recurrence and death in breast cancer patients (25). However, the evidence that MDT care interventions are helpful in patient care is controversial (26, 27). Nonetheless, due to the complexity of HCC treatment, MDT care is emphasized to improve timely and fitting treatment guidelines and the overall survival of HCC patients (28). To improve the quality of cancer care, Taiwan’s Ministry of Health and Welfare (MOHW) promulgated the Cancer Control Act in 2003 and initiated the Complete Cancer Care Quality Improvement Project in 2005, which helped hospitals set up an MDT care meeting for cancer patients. The National Health Insurance (NHI) system is designed with a “Cancer Patient Treatment Planning and Consultation Fee” to encourage the establishment of MDT care plans (29). The Cancer Patient Treatment Planning and Consultation Fee is limited to one declaration for patients with a confirmed cancer or recurrence diagnosis according to the MDT treatment plan. Based on the Cancer Control Act and Regulations for Cancer Care Quality Assurance Measures, medical institutions follow the regulations to establish a committee and assign designated physicians to take charge of the cancer patient care tasks. The medical institutions must follow rigorous standards and pass the hospital evaluation, then get the qualification as a medical institution of cancer control (29). The MDT committee in each hospital abides by the regulations to hold a scheduled meeting with prescribed participating experts. Once a people diagnosed with cancer disease, the doctor may refer the patient to a cancer care team and implement MDT care. The MDT treatment plan was discussed and made based on the consensus of various specialists in the regular meetings. After the MDT meetings, the oncology nurse helps complete the medical record and upload the required information in the NHI system. Details regarding MDT care in Taiwan were described in our previous study (17, 21).

Studies using nationwide populations to explore MDT interventions for HBV- or HCV-associated HCC are lacking. This study aimed to explore the factors associated with MDT care in patients with HCC due to CHB or CHC, and to investigate the treatment outcomes and prognosis of patients with HCC who underwent MDT care. This study can be used as an important reference to improve cancer care and provide resources for health insurance policies.

## 2 Materials and methods

### 2.1 Study design and data sources

This was a retrospective cohort study of a national population. Data was sourced from the population-based Taiwan Cancer Registry Database (TCRD), which records information on all types of cancers diagnosed and treated in Taiwan with excellent quality and high completeness (97%). The records from the TCRD were linked with the Taiwan National Health Insurance Research Database (NHIRD) and the Cause of Death files obtained from the MOHW. The NHIRD is a comprehensive healthcare database that covers almost the entire population (up to 99.99%) of this country (30). This study was reviewed and approved by the Research Ethics Committee of China Medical University and Hospitals in Taichung, Taiwan (IRB number: CMUH110-REC3-227).

### 2.2 Study participants

**Figure 1:**
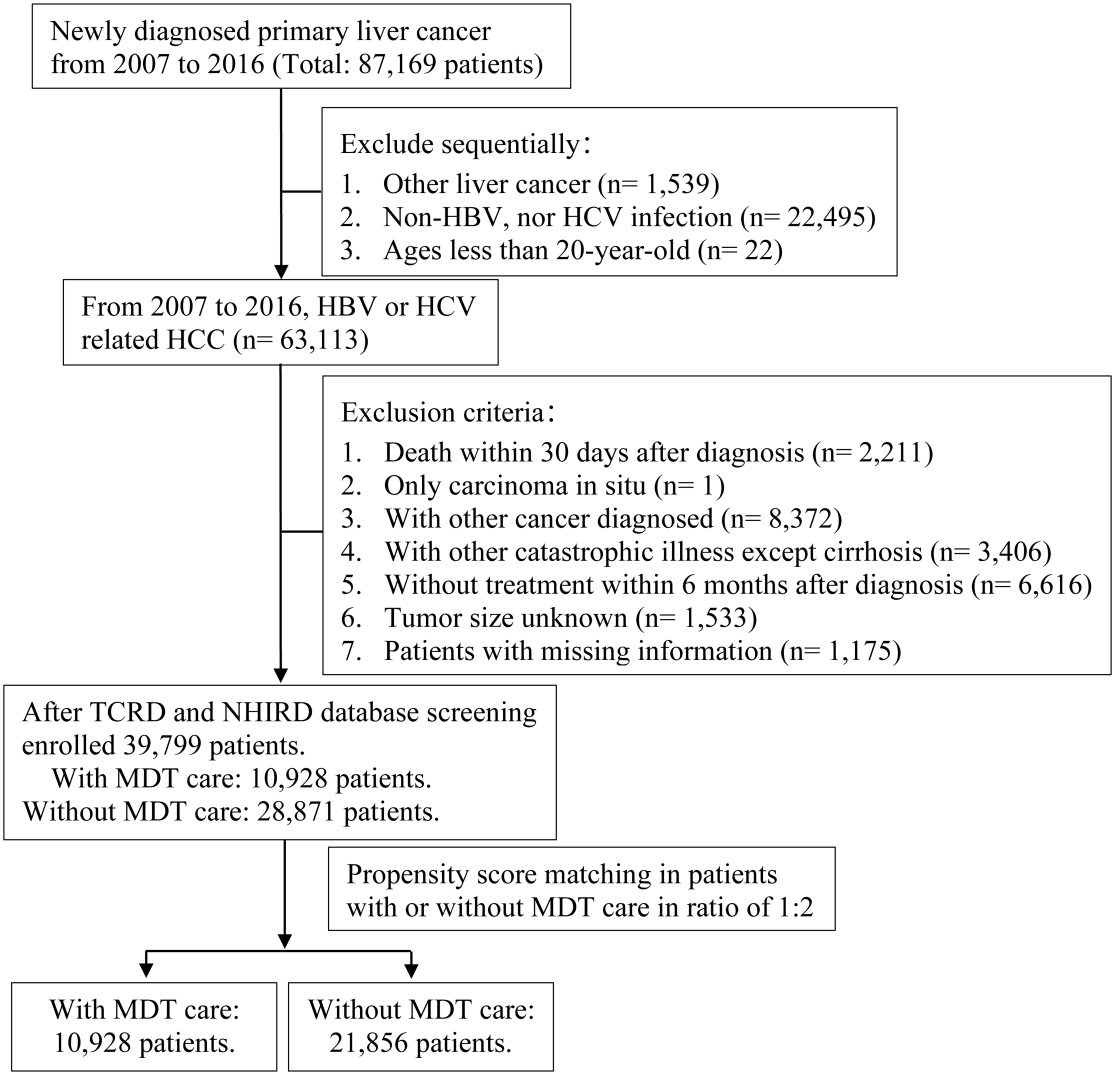
Flowchart of the study participant screening.

First, the authors applied TCRD to select all patients newly diagnosed with HCC from January 1, 2007, to December 31, 2016. We defined the study population as those aged ≥ 20 years with CHB or CHC. This study defined disease status based mainly on diagnosis codes according to the International Classification of Diseases, Ninth Revision, Clinical Modification (ICD-9-CM) or International Classification of Diseases, Tenth Revision, Clinical Modification (ICD-10-CM). The confirmed diagnosis of primary malignant neoplasm of the liver (hepatocellular carcinoma, HCC) was based on ICD-9-CM code:155.0 or ICD-10-CM code C22.0. Those who were infected with CHB used ICD-9-CM codes: 070.20, 070.21, 070.22, 070.23, 070.30, 070.31, 070.32, 070.33, V02.61 or ICD-10-CM codes: B18.0, B18.1, B19.1. Those who were infected with CHC used ICD-9-CM codes: 070.41, 070.44, 070.51, 070.54, 070.70, 070.71, V02.62 or ICD-10-CM codes: B18.2, B19.2, B19.21. This study assessed the survival status of HCC patients who received MDT care. The exclusion criteria were as follows:1) death within 30 days after diagnosis, 2) those who were later confirmed to have carcinoma *in situ*, 3) those diagnosed with other cancers, 4) those with other catastrophic illnesses, except for cirrhosis, 5) those without treatment within six months after diagnosis, and 6) those with missing relevant information. Whether a research subject had joined MDT care was based on the medical record, which declared a Cancer Patient Treatment Planning and Consultation Fee (47079 B). All included patients were followed up until death, loss to follow-up, or December 31, 2018, whichever occurred first. Ultimately, 39,799 patients were enrolled in this study. We used propensity score matching (PSM) to match the group of patients with HCC who received MDT care to those who did not at a ratio of 1:2. A flowchart of the screening process for study participants is shown in **Figure 1**.

### 2.3 Variable definitions and explanations

The typical characteristics of the patients with HCC were also examined. Age was defined as the age at which the study participants had a confirmed diagnosis of HCC. Participants’ socioeconomic status was based on their monthly salaries and grouped according to the National Health Insurance Administration. The environmental factor used was the degree of urbanization of the residential areas. The level of urbanization was established at seven degrees, from highly urbanized areas (level 1) to rural areas (level 7). Patients’ health status included comorbidity severity (Charlson Comorbidity Index [CCI]) and cirrhosis severity. According to the patient’s diagnosis, the comorbidity score of patients within two years before cancer diagnosis was calculated as an indicator of the severity of comorbidities in the study population. CCI was recorded along with Deyo’s Charlson Comorbidity index (31). The severity of cirrhosis was divided into no cirrhosis, mild cirrhosis, and severe cirrhosis in three groups. The confirmed diagnosis of cirrhosis was based on the ICD-9-CM codes 571.5, and ICD-10-CM codes K70.2, K70.30, K70.31, K74.1, K74.60, and K74.69. Patients who had a catastrophic cirrhosis illness belonged to the severe cirrhosis group; patients with only a diagnosis of cirrhosis were grouped into the mild cirrhosis group. Based on the NHI guidelines, the requirements for catastrophic illness of cirrhosis include cirrhosis complicated with 1) massive ascites that cannot be controlled, 2) esophageal varices or gastric varices with bleeding, or 3) hepatic encephalopathy or liver decompensation. This study excluded patients with other catastrophic illnesses because the catastrophic illness of cirrhosis was included in the classification of cirrhosis severity. The definition of ‘catastrophic illness’ aligns with the NHI definition, which includes 30 major disorders such as malignant neoplasm, chronic kidney disease requiring regular hemodialysis, respiratory failure requiring long-term mechanical ventilation, and liver cirrhosis with complications etc. (30). Whether patients received antiviral treatment for CHB or CHC is also an important factor affecting the prognosis of HCC, so the variable of antiviral treatment was divided into two groups: receiving antiviral therapy yes or no. Tumor factors included the tumor size and cancer stage. Tumor size according to the TCRD was recorded based on the maximum diameter of the tumor (if there were multiple nodules in one patient, only the diameter of the largest nodule was registered) and grouped into < 3 cm, 3-5 cm, and > 5 cm. Cancer staging was based on the Barcelona Clinic Liver Cancer (BCLC) classification and divided into BCLC stages 0, A, B, C, D, and unknown. The definitions of applied therapy were based on the relevant treatment codes as stated in the NHIRD, which were cross-comparisons with the therapy registered in the TCRD. A primary medical care facility was defined as a patient’s major care hospital. Medical institutions were divided into medical centers, regional hospitals, and district hospitals. Hospital ownership was allocated to public and nonpublic hospitals.

### 2.4 Outcomes and measurements

This study had two significant outcomes. The first outcome was diagnosis-to-treatment interval (DTI). DTI was defined as the time interval from the date of a confirmed diagnosis of HCC (date of imaging study or liver biopsy) to the date of the first course of treatment (surgery, local treatment, embolization, radiotherapy, or chemotherapy). Patients with HCC generally undergo an imaging assessment of therapy within three–six months of the first treatment. To clearly define the time frame of the first course of treatment, the treatment combination was defined as within six months after the confirmed diagnosis of HCC (32). The second outcome was cancer-related death. Cancer-related death was based on patient data from the Cause of Death file and compared with the NHIRD for validation.

### 2.5 Statistical analysis

Descriptive statistics were used to evaluate the univariate association between various variables (**Table 1**) and the status of participation in MDT care for patients with HCC. Statistics such as the number and percentage of each variable were calculated. DTI was expressed as the median and quartile.

**Table 1:** Bivariate analysis and multivariate logistic regression analysis of hepatocellular carcinoma patient characteristics with or without multidisciplinary team care.

Variables	Total		Non-MDT		MDT		*p value^a^*	Adjusted			*p value^b^*
	N	%	n1	%	n2	%		OR	95% CI		
Total	39799	100.00	28871	72.54	10928	27.46					
Sex							0.021				
Female	11200	28.14	8218	73.38	2982	26.63		1.00			
Male	28599	71.86	20653	72.22	7946	27.78		1.02	0.97	1.07	0.493
Age at the time of diagnosis (years)							<0.001				
≦ 44	3086	7.75	2244	72.72	842	27.28		1.00			
45-54	7286	18.31	5199	71.36	2087	28.64		1.06	0.96	1.17	0.239
55-64	12259	30.80	8836	72.08	3423	27.92		1.02	0.93	1.12	0.668
65-74	10912	27.42	7931	72.68	2981	27.32		1.00	0.90	1.10	0.916
≧ 75	6256	15.72	4661	74.50	1595	25.50		0.85	0.76	0.94	0.002
Average age (mean ± SD)	62.01 ± 11.93		62.11 ± 11.96		61.74 ± 11.84		0.006				
Monthly salary (NTD)							<0.001				
≦ 20,008	3428	8.61	2612	76.20	816	23.80		1.00			
20,009-22,800	15227	38.26	10998	72.23	4229	27.77		1.07	0.97	1.17	0.175
22,801-28,800	8571	21.54	6244	72.85	2327	27.15		1.00	0.90	1.10	0.919
28,801-36,300	3438	8.64	2504	72.83	934	27.17		1.00	0.89	1.12	0.989
36,301-45,800	4648	11.68	3292	70.83	1356	29.17		1.08	0.97	1.20	0.152
≧ 45,801	4487	11.27	3221	71.79	1266	28.21		1.06	0.96	1.18	0.262
Urbanization level							<0.001				
Level 1	9412	23.65	6741	71.62	2671	28.38		1.00			
Level 2	11184	28.10	8020	71.71	3164	28.29		0.99	0.93	1.06	0.850
Level 3	6413	16.11	4866	75.88	1547	24.12		0.82	0.76	0.88	<0.001
Level 4	6816	17.13	4866	71.39	1950	28.61		1.04	0.97	1.12	0.313
Level 5-7	5974	15.01	4378	73.28	1596	26.72		0.92	0.85	1.00	0.049
Charlson Comorbidity Index							<0.001				
0	2293	5.76	1578	68.82	715	31.18		1.00			
1	8444	21.22	5945	70.41	2499	29.59		1.00	0.91	1.11	0.944
2	6214	15.61	4438	71.42	1776	28.58		0.98	0.87	1.09	0.657
≧ 3	22848	57.41	16910	74.01	5938	25.99		0.93	0.84	1.03	0.167
Severity of cirrhosis							<0.001				
No cirrhosis	21372	53.70	15061	70.47	6311	29.53		1.00			
Mild cirrhosis	16605	41.72	12355	74.41	4250	25.59		0.85	0.81	0.89	<0.001
Severe cirrhosis	1822	4.58	1455	79.86	367	20.14		0.66	0.58	0.75	<0.001
Tumor size (centimeters)							0.003				
< 3	15859	39.85	11615	73.24	4244	26.76		1.00			
3-5	9900	24.87	7212	72.85	2688	27.15		1.05	0.98	1.11	0.147
> 5	14040	35.28	10044	71.54	3996	28.46		1.09	1.02	1.17	0.018
Cancer stage – BCLC stage							<0.001				
0	2639	6.63	1824	69.12	815	30.88		1.00			
A	13626	34.24	9553	70.11	4073	29.89		0.89	0.81	0.98	0.021
B	8000	20.10	5536	69.20	2464	30.80		0.88	0.79	0.98	0.024
C	7342	18.45	5216	71.04	2126	28.96		0.84	0.75	0.94	0.003
D	822	2.07	662	80.54	160	19.46		0.53	0.43	0.65	<0.001
Unknown	7370	18.52	6080	82.50	1290	17.50		0.46	0.41	0.52	<0.001
Hospital level							<0.001				
Medical centers	25021	62.87	18968	75.81	6053	24.19		1.00			
Regional hospitals	14606	36.70	9765	66.86	4841	33.14		1.96	1.87	2.06	<0.001
District hospitals	172	0.43	138	80.23	34	19.77		1.03	0.71	1.51	0.866
Hospital ownership							<0.001				
Public	12324	30.97	7853	63.72	4471	36.28		1.00			
Non-public	27475	69.03	21018	76.50	6457	23.50		0.45	0.43	0.48	<0.001

BCLC, Barcelona Clinic Liver Cancer classification; CI, confidence index; MDT, multidisciplinary team; NTD, New Taiwan Dollar; OR, odds ratio; SD, standard deviation.

a Chi-square tests.

b Multivariate logistic regression analysis.

The study used variables, including sex, age at diagnosis, comorbidity severity (CCI), cirrhosis severity, antiviral therapy, tumor size, and tumor staging, to build a logistic regression model to calculate the propensity score for matching. Then, the propensity score using greedy nearest neighbor matching by digit without replacement was used to form a focus matching set to match the group of HCC patients who joined MDT care to those who did not receive MDT at a ratio of 1:2 to mitigate selection bias. The MDT and non-MDT patients have been matched in the same diagnosis year. The process executed the “best” match first, then the “next best” match in a classified sequence until no more matches could be made. Those with the highest digit match in the propensity score were considered the best matches. Each control sample was selected only once. The final matched-pair tests comprised tightly matched individual pairs and balanced control and case groups.

The Chi-square test was used to investigate whether patients with HCC were involved in MDT care and patient demographic characteristics (sex and age), socioeconomic status (monthly salary), urbanization level of the residence area, health condition, tumor characteristics (tumor size and cancer stage), and hospital characteristics (hospital level and hospital ownership). As DTI was not normally distributed, non-parametric statistics with the Kruskal-Wallis H test and Wilcoxon rank-sum test were used to analyze differences in DTI between patients with HBV- or HCV-induced HCC with or without MDT care intervention. The DTI time was then transformed into a natural log for further analysis. Multiple regression models with Generalized Estimating Equations were used for multivariate analysis to determine whether MDT care affected DTI. The results are presented as ratios with 95% confidence interval.

The log-rank test was employed as a bivariate analysis to evaluate the risk factors for cancer death after being involved in MDT. For the matched groups, the stratified Cox proportional hazards model was used to examine whether MDT care influenced the risk of death in patients with HCC when individual characteristics, socioeconomic status, environmental condition, health status, tumor features, and hospital characteristics were controlled (33). The results are presented as hazard ratios and 95% confidence intervals (CIs). The authors composed the survival curve of HCC patients with MDT versus non-MDT involvement at different BCLC stages after controlling for confounding variables, as listed above. All statistical analyses were performed using the SAS software (version 9.4, SAS Institute Inc., Cary, NC, USA). All tests were two-sided, and the level of statistical significance was set at *p* < 0.05.

## 3 Results

After participant enrollment, the total number of study participants was 39,799. A total of 10,928 patients with HBV- or HCV-related HCC underwent MDT (**Table 1**). Before matching, bivariate analysis showed significant differences in demographic characteristics (sex and age), socioeconomic factors (monthly salary), environmental factors (urbanization level), patient health status (CCI and severity of cirrhosis), tumor characteristics (tumor size, cancer stage), and medical institution characteristics (hospital level, hospital ownership) between patients with HCC participating in and those not participating in MDT (*p* < 0.05) (**Table 1**). After multivariate adjustment, HCC patients diagnosed at ≥ 75 years of age were 15% less likely to receive MDT care than those diagnosed at ≤ 44 years of age (OR 0.85, 95% CI 0.76-0.94). Increasing severity of cirrhosis and HCC stage at the time of diagnosis were associated with lower odds of receiving MDT care. However, increasing tumor size at diagnosis was associated with higher odds of receiving MDT care. Compared with those visiting medical centers, patients visiting regional hospitals were around two times more likely to receive MDT care. Patients at non-public hospitals were less likely to receive MDT (**Table 1**).

After PSM, 32,784 patients were enrolled in this study. After matching, bivariate analysis showed no significant differences in sex, age, monthly salary, CCI, severity of cirrhosis, antiviral therapy, tumor size, and cancer stage between the two groups (*p* > 0.05) (**Table 2**).

**Table 2:** Bivariate analysis of factors associated with HCC patients participating in MDT post-propensity score matching.

Variables	Post 2:1 matching						
	Total		Non-MDT		MDT		*p* value^a^
	N	%	n1	%	n2	%	
Total	32784	100.00	21856	66.67	10928	33.33	
Sex							0.948
Female	8937	27.26	5955	27.25	2982	27.29	
Male	23847	72.74	15901	72.75	7946	72.71	
Age at the time of diagnosis (years)							0.865
≦ 44	2509	7.65	1667	7.63	842	7.70	
45-54	6198	18.90	4111	18.81	2087	19.10	
55-64	10325	31.49	6902	31.58	3423	31.32	
65-74	9026	27.53	6045	27.66	2981	27.28	
≧ 75	4726	14.42	3131	14.33	1595	14.60	
Average age (mean ± SD)			61.72 ± 11.74		61.74 ± 11.84		0.853^b^
Monthly salary (NTD)							0.446
≦ 20,008	2515	7.67	1699	7.77	816	7.47	
20,009-22,800	12547	38.27	8318	38.06	4229	38.70	
22,801-28,800	7116	21.71	4789	21.91	2327	21.29	
28,801-36,300	2864	8.74	1930	8.83	934	8.55	
36,301-45,800	3981	12.14	2625	12.01	1356	12.41	
≧ 45,801	3761	11.47	2495	11.42	1266	11.58	
Urbanization level							<0.001
Level 1	7768	23.69	5097	23.32	2671	24.44	
Level 2	9308	28.39	6144	28.11	3164	28.95	
Level 3	5226	15.94	3679	16.83	1547	14.16	
Level 4	5592	17.06	3642	16.66	1950	17.84	
Level 5-7	4890	14.92	3294	15.07	1596	14.60	
Charlson Comorbidity Index							0.347
0	2052	6.26	1337	6.12	715	6.54	
1	7416	22.62	4917	22.50	2499	22.87	
2	5338	16.28	3562	16.30	1776	16.25	
≧ 3	17978	54.84	12040	55.09	5938	54.34	
Severity of cirrhosis							0.197
No cirrhosis	18726	57.12	12415	56.80	6311	57.75	
Mild cirrhosis	12975	39.58	8725	39.92	4250	38.89	
Severe cirrhosis	1083	3.30	716	3.28	367	3.36	
History of anti-virus therapy							0.246
No	16926	51.63	11334	51.86	5592	51.17	
Yes	15858	48.37	10522	48.14	5336	48.83	
Tumor size (centimeters)							0.269
< 3	12922	39.42	8678	39.71	4244	38.84	
3-5	7933	24.20	5245	24.00	2688	24.60	
> 5	11929	36.39	7933	36.30	3996	36.57	
Cancer stage – BCLC stage							0.774
0	2475	7.55	1660	7.60	815	7.46	
A	12300	37.52	8227	37.64	4073	37.27	
B	7281	22.21	4817	22.04	2464	22.55	
C	6386	19.48	4260	19.49	2126	19.45	
D	448	1.37	288	1.32	160	1.46	
Unknown	3894	11.88	2604	11.91	1290	11.80	
Hospital level							<0.001
Medical centers	20328	62.01	14275	65.31	6053	55.39	
Regional hospitals	12319	37.76	7478	34.21	4841	44.30	
District hospitals	137	0.42	103	0.47	34	0.31	
Hospital ownership							<0.001
Public	10447	31.87	5976	27.34	4471	40.91	
Non-public	22337	68.13	15880	72.66	6457	59.09	

BCLC, Barcelona Clinic Liver Cancer classification; MDT, multidisciplinary team; NTD, New Taiwan Dollar.

a Chi-square tests.

b paired t-test.

HCC patients who had participated in MDT care had a longer DTI than those who did not participate in MDT care (median:22 days vs. 20 days, *p* < 0.05) (**Supplementary Table 1**). Patients with no comorbidities or without cirrhosis had the shortest DTI. DTI was shorter in patients with larger tumors or more advanced tumors but was longer among patients who visited medical centers and public hospitals (**Supplementary Table 1**). We compared the DTI of liver cancer patients with different characteristics between MDT and non-MDT care groups. This revealed that patients with HCC who participated in MDT care had longer DTI for the most relevant factors. The average day of DTI was 29.55 ± 28.93 days in MDT care participants compared to 27.91 ± 29.65 days in non-MDT care participants (**Supplementary Table 2**). After controlling for the relevant variables, DTI increased by 1.24 times for HCC patients with MDT care than for those without MDT care (ratio = 1.24, 95% CI:1.18-1.32) (**Table 3**).

**Table 3:** Factors associated with diagnosis to the first treatment time interval in patients with HCC.

Variables	Adjusted model ^a^			
	Ratio	95% CI		*p* value
MDT				
Non-participants (Reference)				
Participants	1.24	1.18	1.32	<0.001
Sex				
Female (Reference)				
Male	0.93	0.88	0.99	0.029
Age at the time of diagnosis (years)				
≦ 44 (Reference)				
45-54	1.23	1.08	1.38	0.001
55-64	1.38	1.23	1.55	<0.001
65-74	1.38	1.22	1.55	<0.001
≧ 75	1.29	1.13	1.48	<0.001
Monthly salary (NTD)				
≦ 20,008 (Reference)				
20,009-22,800	1.09	0.97	1.22	0.161
22,801-28,800	1.08	0.95	1.22	0.239
28,801-36,300	1.13	0.99	1.30	0.075
36,301-45,800	1.07	0.94	1.22	0.322
≧ 45,801	1.08	0.95	1.23	0.237
Urbanization level				
Level 1 (Reference)				
Level 2	0.96	0.89	1.03	0.288
Level 3	0.93	0.85	1.02	0.116
Level 4	0.91	0.83	1.00	0.040
Level 5-7	1.00	0.91	1.10	0.945
Charlson Comorbidity Index				
0 (Reference)				
1	1.40	1.23	1.59	<0.001
2	1.47	1.29	1.68	<0.001
≧ 3	1.62	1.43	1.83	<0.001
Severity of cirrhosis				
No cirrhosis (Reference)				
Mild cirrhosis	0.61	0.58	0.65	<0.001
Severe cirrhosis	0.77	0.65	0.91	0.002
History of anti-virus therapy				
No (Reference)				
Yes	1.08	1.02	1.14	0.011
Tumor size (centimeters)				
< 3 (Reference)				
3-5	0.70	0.66	0.75	<0.001
> 5	0.33	0.30	0.36	<0.001
Cancer stage – BCLC stage				
0 (Reference)				
A	0.79	0.73	0.85	<0.001
B	0.60	0.54	0.67	<0.001
C	0.51	0.45	0.58	<0.001
D	0.34	0.24	0.47	<0.001
Unknown	0.42	0.38	0.48	<0.001
Hospital level				
Medical centers (Reference)				
Regional hospitals	0.73	0.68	0.77	<0.001
District hospitals	0.65	0.40	1.07	0.089
Hospital ownership				
Public (Reference)				
Non-public	0.88	0.83	0.94	<0.001

BCLC, Barcelona Clinic Liver Cancer classification; CI, confidence index; MDT, multidisciplinary team; NTD,New Taiwan Dollar.

a Multiple regression analysis with generalized estimating equations: The natural logarithmic conversion was taken from the diagnosis to the first treatment interval, and the analysis result coefficients were then used to extract the values after the exponential conversion.

A log-rank test was used to analyze which factors were related to the decreased risk of cancer-related death in HBV- or HCV-related HCC patients after joining MDT care (**Supplementary Table 3**). By the end of the study, the total number of deaths among patients with HCC was 5,904 (54.03%) and 11,203 (51.26%) in the MDT and non-MDT care groups, respectively. However, the mortality of patients with HCC who did or did not participate in MDT care was not statistically significant (*p* = 0.206). Patients who lived in urbanization level 3 and received MDT care had a lower death rate than those who did not participate in MDT (51.58% vs. 52.49%, *p* = 0.034). Patients who visited medical centers (Death: MDT vs. non-MDT= 48.22% vs. 50.51%, *p* < 0.001) or public hospitals (Death: MDT vs. non-MDT= 48.62% vs. 50.87%, *p* = 0.002) with MDT care had a relatively lower death rate than those who did not (**Supplementary Table 3**).

After multivariate adjustment, patients who received MDT care had 12% higher survival than those who did not (HR=0.88, 95% CI:0.84-0.92) (**Table 4**). The receipt of anti-viral therapy for HBV- or HCV-related HCC was also associated with higher survival (lower risk of death). Increasing tumor size or a higher HCC stage were both independently associated with a higher risk of death (**Table 4** and **Supplementary Figure 1**). HCC patients who received only surgical intervention or treatment combination with surgery within six months after diagnosis had better survival conditions (lower HR of cancer death) (**Table 4**).

**Table 4:** Analysis of MDT participant status and various factors affecting cancer death in HCC patients.

Variables	Total		Censored		Event		*p* value^a^	Adjusted				*p* value^b^
	N	%	n1	%	n2	%		HR		95% CI		
Total	32784	100.00	15677	47.82	17107	52.18						
MDT							0.206					
Non-participants	21856	66.67	10653	48.74	11203	51.26						
Participants	10928	33.33	5024	45.97	5904	54.03		0.88		0.84	0.92	<0.001
Sex							<0.001					
Female	8937	27.26	4547	50.88	4390	49.12						
Male	23847	72.74	11130	46.67	12717	53.33		1.06		0.95	1.17	0.297
Age at the time of diagnosis (years)							<0.001					
≦ 44	2509	7.65	1130	45.04	1379	54.96						
45-54	6198	18.91	2985	48.16	3213	51.84		0.62		0.52	0.74	<0.001
55-64	10325	31.49	5338	51.70	4987	48.30		0.67		0.57	0.79	<0.001
65-74	9026	27.53	4241	46.99	4785	53.01		0.70		0.59	0.83	<0.001
≧ 75	4726	14.42	1983	41.96	2743	58.04		0.97		0.80	1.18	0.741
Monthly salary (NTD)							<0.001					
≦ 20,008	2515	7.67	1013	40.28	1502	59.72						
20,009-22,800	12547	38.27	5689	45.34	6858	54.66		1.07		0.97	1.17	0.193
22,801-28,800	7116	21.71	3323	46.70	3793	53.30		1.08		0.97	1.19	0.145
28,801-36,300	2864	8.74	1425	49.76	1439	50.24		1.06		0.94	1.20	0.352
36,301-45,800	3981	12.14	2113	53.08	1868	46.92		0.96		0.86	1.07	0.453
≧ 45,801	3761	11.47	2114	56.21	1647	43.79		0.94		0.83	1.05	0.277
Urbanization level							<0.001					
Level 1	7768	23.69	3851	49.58	3917	50.42						
Level 2	9308	28.39	4561	49.00	4747	51.00		0.98		0.92	1.05	0.640
Level 3	5226	15.94	2497	47.78	2729	52.22		0.98		0.91	1.07	0.696
Level 4	5592	17.06	2550	45.60	3042	54.40		0.97		0.89	1.05	0.436
Level 5-7	4890	14.92	2218	45.36	2672	54.64		0.98		0.90	1.07	0.692
Charlson Comorbidity Index							<0.001					
0	2052	6.26	1333	64.96	719	35.04						
1	7416	22.62	4494	60.60	2922	39.40		1.21		1.01	1.44	0.034
2	5338	16.28	3186	59.69	2152	40.31		1.34		1.11	1.61	0.003
≧ 3	17978	54.84	6664	37.07	11314	62.93		2.38		1.99	2.85	<0.001
Severity of cirrhosis							<0.001					
No cirrhosis	18726	57.12	9083	48.50	9643	51.50						
Mild cirrhosis	12975	39.58	6113	47.11	6862	52.89		1.72		1.48	2.01	<0.001
Severe cirrhosis	1083	3.30	481	44.41	602	55.59		2.61		1.60	4.25	<0.001
History of anti-virus therapy							<0.001					
No	16926	51.63	6996	41.33	9930	58.67						
Yes	15858	48.37	8681	54.74	7177	45.26		0.47		0.41	0.54	<0.001
Tumor size (centimeters)							<0.001					
< 3	12922	39.42	8267	63.98	4655	36.02						
3-5	7933	24.20	4160	52.44	3773	47.56		1.23		1.07	1.41	0.003
> 5	11929	36.39	3250	27.24	8679	72.76		1.68		1.43	1.98	<0.001
Cancer stage – BCLC stage							<0.001					
0	2475	7.55	2032	82.10	443	17.90						
A	12300	37.52	7999	65.03	4301	34.97		2.17		1.71	2.76	<0.001
B	7281	22.21	2881	39.57	4400	60.43		4.14		3.17	5.41	<0.001
C	6386	19.48	1196	18.73	5190	81.27		10.52		7.82	14.15	<0.001
D	448	1.37	101	22.54	347	77.46		42.12		21.29	83.34	<0.001
Unknown	3894	11.88	1468	37.70	2426	62.30		16.51		8.03	33.92	<0.001
Treatment							<0.001					
Surgery	9197	28.05	6692	72.76	2505	27.24						
Embolization	2096	6.39	677	32.30	1419	67.70		2.88		2.58	3.22	<0.001
Radiotherapy	1334	4.07	197	14.77	1137	85.23		5.38		4.69	6.16	<0.001
Surgery + local treatment	5764	17.58	3807	66.05	1957	33.95		1.87		1.70	2.06	<0.001
Surgery + embolization	2082	6.35	778	37.37	1304	62.63		2.46		2.20	2.75	<0.001
Embolization + chemotherapy	5414	16.51	1496	27.63	3918	72.37		3.25		2.98	3.54	<0.001
Embolization + radiotherapy + chemotherapy	897	2.74	131	14.60	766	85.40		3.51		3.03	4.07	<0.001
Surgery + embolization + chemotherapy	1376	4.20	496	36.05	880	63.95		2.28		2.01	2.59	<0.001
Surgery + Local treatment + embolization	1079	3.29	513	47.54	566	52.46		2.12		1.83	2.46	<0.001
Surgery + Local treatment + embolization + chemotherapy	1036	3.16	459	44.31	577	55.69		2.30		1.98	2.67	<0.001
Others treatment combination	2509	7.65	431	17.18	2078	82.82		4.34		3.88	4.84	<0.001
Hospital level							<0.001					
Medical centers	20328	62.01	10199	50.17	10129	49.83						
Regional hospitals	12319	37.58	5411	43.92	6908	56.08		1.08		1.02	1.14	0.005
District hospitals	137	0.42	67	48.91	70	51.09		1.16		0.79	1.71	0.452
Hospital ownership							<0.001					
Public	10447	31.87	5233	50.09	5214	49.91						
Non-public	22337	68.13	10444	46.76	11893	53.24		0.96		0.91	1.01	0.138

Event: cancer death.

BCLC, Barcelona Clinic Liver Cancer classification; CI, confidence index; HR, hazard ratio; MDT, multidisciplinary team; NTD, New Taiwan Dollar.

p value^a^: log-rank test analysis.

p value^b^: Conditional Cox proportional hazard model.

**Figure 2:**
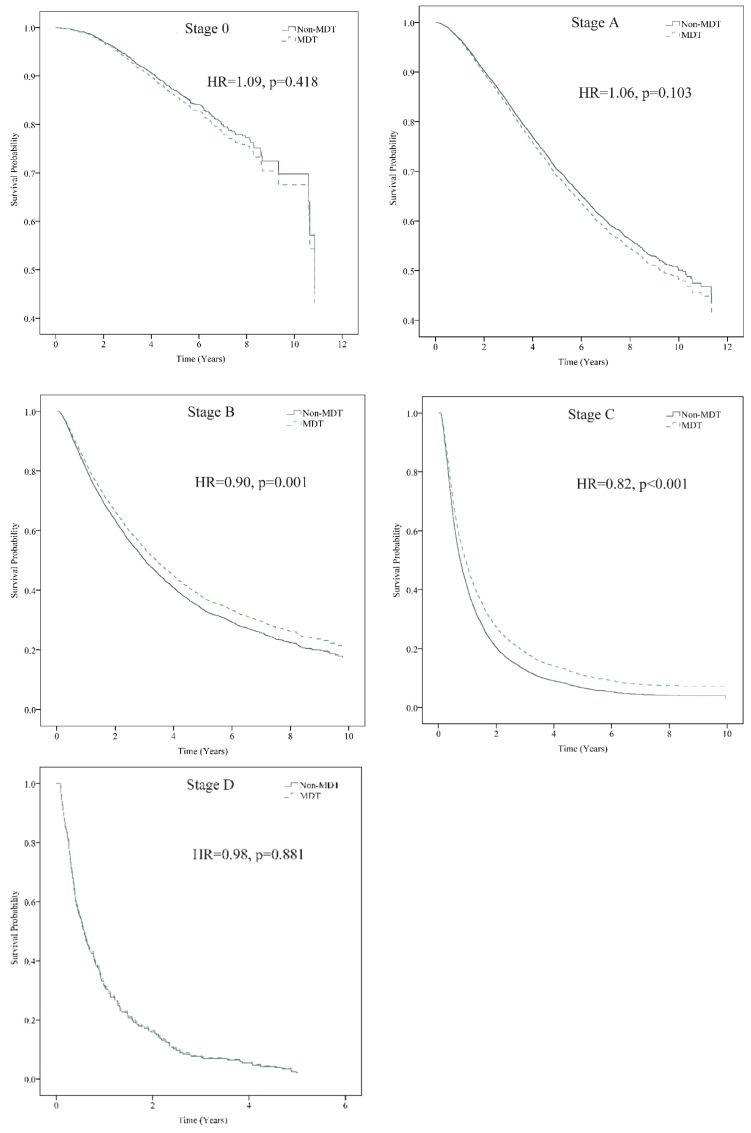
Stratification analysis of the patients according to BCLC stage. MDT care significantly reduced the risk of death for patients with BCLC stage B (Adj. HR = 0.90, p = 0.001) and stage C (Adj. HR = 0.82, p < 0.001) HCC. Event: cancer death. Stratification analysis was controlled for sex, age, monthly salary, urbanization level, CCI, cirrhosis severity, anti-viral therapy, tumor size, treatment method, hospital level, and hospital ownership. Adj. HR, adjusted hazard ratio; BCLC, Barcelona Clinic Liver Cancer classification; HCC, Hepatocellular carcinoma; MDT, multidisciplinary team. *The non-MDT group was the reference group. Cox proportional Hazard model.

On stratified analysis by the BCLC staging system for HCC, after controlling for all other variables, patients with Stage B who received MDT care had a 10% higher survival, and those with Stage C had a 18% higher survival than those without MDT care (**Figure 2**).

## 4 Discussion

In this national population-based study with more than 10 years of coverage, we found that older patients, those with cirrhosis, a later stage of HCC, medical center visits, and non-public hospital visits were less likely to receive MDT care. Patients with HBV- or HCV-related HCC who had MDT care had prolonged DTI, but higher survival, while those with stages B and C also had a lower risk of cancer-related death (higher survival) when they received MDT care.

Yegin et al. compared surgical versus non-surgical treatment of HCC and found that treatment decisions were becoming more complex for increasing diversity and availability of treatment options. Therefore, comprehensive MDT care is required for HCC treatment (34). MDT care integrates the recommendations of medical experts to improve cancer care quality. However, this study found that old age, cirrhosis, late cancer stage, treatment in medical centers, and non-public hospitals were all factors contributing to a lower chance of participating in MDT care. Although the treatment options for HCC are varied and complex, this cancer staging system helps clinicians easily make treatment choices for HCC. The Taiwan Liver Cancer Association and Gastroenterological Society of Taiwan recommend using the BCLC staging system as the primary cancer staging and treatment guidance system (35). In Taiwan, hospital cancer care accreditation may compel hospitals, particularly medical centers and regional hospitals, to implement MDT care. Most district hospitals are non-public hospitals with limited capacity to implement MDT care.

After adjusting for correlated factors, patients with HBV- or HCV-related HCC who participated in MDT care had a longer time interval from diagnosis to first treatment than those who did not participate in MDT care. Studies have reported that demographic and socioeconomic factors may affect DTI (36, 37). Sharma et al. further pointed out that treatment modalities (radiation therapy) in medical centers and hospitals with large treatment volumes will extend DTI (37). Some researchers have shown that prolonged DTI is associated with poor cancer prognosis (37–40). However, clinical studies have reported no significant correlation between longer DTI and local tumor control, survival without distal metastasis, or overall survival in head and neck cancer (41). A systemic review article concluded that no significant association exists between longer DTI and poor prognosis in colorectal cancer (42). One population-based study revealed that early-stage HCC patients (AJCC stage I and II) who have prolonged DTI (> 60 days) would have lower survival rates (43). Taiwan’s MOHW has promulgated multiple rules to incentivize clinicians and people to strengthen the tracking and treatment of viral hepatitis. In a previous study in Taiwan, 78.46% of patients with HCC received early-stage cancer therapy within 30 days of diagnosis (43). Therefore, when MDT care requires regular meetings, the DTI is prolonged. In this study, patients with HBV- or HCV-related HCC who participated in MDT care had prolonged DTI compared to those who did not.

Several studies have reported that MDT care in patients with cancer has an improved survival rate or a decreased risk of cancer-related death (10, 15, 17–21, 24, 25). MDT care is used in patients with HCC to assist clinicians in making accurate diagnoses and treatment choices and prolonging or improving survival status (28, 44–48). In addition, studies have shown that MDT care increases the proportion of patients with HCC receiving appropriate therapy (5, 44–46, 49, 50). This nationwide population-based cohort study also found that HBV- and HCV-related HCC patients had a reduced risk of death after participating in MDT care. According to Chen et al., compared with the AJCC staging system, the BCLC staging system has a better long-term prognostic prediction for curative therapies, such as surgical treatment, in HCC patients (51). Most of the participants in this study whose therapy combination included surgery (62.73%) as the first treatment after diagnosis. Therefore, the BCLC staging system in this study has a better prognostic prediction than the AJCC staging system, which is commonly used for other cancers. Sinn et al. conducted a cohort study that enrolled 6,619 newly diagnosed HCC patients over nine years. It was concluded that the subgroup of patients with poor liver function (albumin-bilirubin grade 2 or 3), high alpha-fetoprotein (≥ 200 ng/mL), and intermediate to advanced HCC (BCLC stage B or C) who received MDT care had specific improvements in survival benefits (47). This study confirmed that HCC patients with BCLC stage B or C who participated in MDT care had a significantly higher survival rate.

This study used a nationwide population-based research database and PSM to eliminate selection bias; therefore, the sample was representative. However, the present study has some limitations. First, secondary data were used, and some personal information about detailed cancer treatment and disease prognosis could not be obtained. Second, some important clinical data influencing outcomes, such as portal hypertension, laboratory data, detailed Child-Turcotte-Pugh score, accurate tumor numbers, and each tumor’s actual size, were unavailable. This study employed cirrhosis severity to compensate for data limitations in assessing accurate liver function. Third, the members of the MDT (the actual members of medical experts), the method (direct meeting or virtual meeting), and the period of MDT meetings (the accurate frequency of MDT meetings, usually once a week in Taiwan) were different in every hospital; therefore, the study populations joining the exact model of MDT care were unknown. Even though there is a minor difference in MDT care in each hospital, the medical institute must follow the Act and Regulations to hold MDT conferences, maintain cancer care quality, and get cancer care qualifications. Fourth, we did not match variables that had less relation to disease outcome, such as urbanization level, hospital level, and hospital ownership. However, we have included those variables in the multivariate model to control them.

In conclusion, patients with HBV- or HCV-related HCC who participated in MDT care had longer DTI but a lower risk of cancer death. In patients with intermediate-to advanced-stage HCC (BCLC stage B or C), participation in MDT care significantly improved their outcomes. Hospitals should provide HCC patients with multidisciplinary team care to improve cancer care.

## Data availability statement

The raw data supporting the conclusions of this article will be made available by the authors, without undue reservation.

## Ethics statement

The research was conducted in accordance with the 1964 Declaration of Helsinki and its amendments and was reviewed and approved by the Research Ethics Committee of China Medical University and Hospitals, Taichung, Taiwan (IRB number: CMUH110-REC3-227). The need for informed consent was waived due to the use of anonymized secondary data.

## Author contributions

Y-CT, C-YP, and W-CT contributed to the conception and design of the study. W-YC, P-TK, and W-CT collected the database and performed the statistical analysis. Y-CT and P-TK wrote the manuscript. All authors contributed to the manuscript revision read, and approved the submitted version.
