# Supplementary material for: Effect of multidisciplinary team care on patient survival in chronic hepatitis B or C hepatocellular carcinoma
Source: Front Oncol. 2023 Dec 21;13:1251571. doi: 10.3389/fonc.2023.1251571 (PMC10764426; doi:10.3389/fonc.2023.1251571)
Supplement: Supplementary file 1 [file DataSheet_1.docx]

**Supplementary material**

Supplementary Table 1 Bivariate analysis of the time interval from diagnosis to first treatment in HCC patients after 1:2 matching for each variable.

| **Variables** | N | Mean | SD | Median | Quartile | | *p* value ^a^ |
| --- | --- | --- | --- | --- | --- | --- | --- |
|  |  |  |  |  | Q1 | Q3 |  |
| **Total** | 32784 | 28.45 | 29.42 | 21 | 8 | 38 |  |
| **MDT** | | | | | | | <0.001 ^b^ |
| Non-participants | 21856 | 27.91 | 29.65 | 20 | 7 | 38 |  |
| Participants | 10928 | 29.55 | 28.93 | 22 | 10 | 40 |  |
| **Sex** | | | | | | | <0.001 ^b^ |
| Female | 8937 | 31.18 | 30.86 | 23 | 10 | 42 |  |
| Male | 23847 | 27.43 | 28.80 | 20 | 7 | 37 |  |
| **Age at the time of diagnosis (years)** | | | | | | | <0.001 |
| ≦ 44 | 2509 | 22.19 | 25.84 | 15 | 5 | 30 |  |
| 45-54 | 6198 | 26.39 | 28.40 | 19 | 7 | 35 |  |
| 55-64 | 10325 | 29.44 | 29.99 | 21 | 9 | 40 |  |
| 65-74 | 9026 | 29.61 | 29.51 | 22 | 9 | 40 |  |
| ≧ 75 | 4726 | 30.11 | 30.50 | 22 | 8 | 41 |  |
| **Monthly salary (NTD)** | | | | | | | <0.001 |
| ≦ 20,008 | 2515 | 26.72 | 30.06 | 18 | 6 | 36 |  |
| 20,009-22,800 | 12547 | 28.75 | 29.64 | 21 | 8 | 39 |  |
| 22,801-28,800 | 7116 | 29.19 | 30.21 | 21 | 8 | 40 |  |
| 28,801-36,300 | 2864 | 28.31 | 29.00 | 20 | 9 | 38 |  |
| 36,301-45,800 | 3981 | 28.07 | 28.15 | 21 | 9 | 38 |  |
| ≧ 45,801 | 3761 | 27.73 | 28.30 | 21 | 8 | 36 |  |
| **Urbanization level** | | | | | | | 0.0053 |
| Level 1 | 7768 | 29.02 | 29.38 | 21 | 9 | 40 |  |
| Level 2 | 9308 | 28.56 | 29.53 | 21 | 8 | 39 |  |
| Level 3 | 5226 | 27.69 | 28.49 | 20 | 8 | 38 |  |
| Level 4 | 5592 | 27.83 | 29.46 | 20 | 7 | 37 |  |
| Level 5-7 | 4890 | 28.88 | 30.18 | 21 | 8 | 39 |  |
| **Charlson Comorbidity Index** | | | | | | | <0.001 |
| 0 | 2052 | 21.28 | 23.17 | 15 | 5 | 29 |  |
| 1 | 7416 | 26.58 | 27.64 | 19 | 8 | 35.5 |  |
| 2 | 5338 | 28.74 | 28.99 | 21 | 9 | 38 |  |
| ≧ 3 | 17978 | 29.96 | 30.71 | 22 | 8 | 41 |  |
| **Severity of cirrhosis** | | | | | | | <0.001 |
| No cirrhosis | 18726 | 25.94 | 26.65 | 19 | 8 | 35 |  |
| Mild cirrhosis | 12975 | 31.38 | 32.20 | 23 | 8 | 43 |  |
| Severe cirrhosis | 1083 | 36.95 | 34.88 | 28 | 13 | 51 |  |
| **History of anti-virus therapy** | | | | | | | <0.001 ^b^ |
| No | 16926 | 27.25 | 29.06 | 19 | 7 | 37 |  |
| Yes | 15858 | 29.74 | 29.74 | 22 | 9 | 40 |  |
| **Tumor size (centimeters)** | | | | | | | <0.001 |
| < 3 | 12922 | 35.87 | 31.67 | 28 | 14 | 47 |  |
| 3-5 | 7933 | 29.46 | 28.76 | 22 | 10 | 40 |  |
| > 5 | 11929 | 19.75 | 24.62 | 13 | 3 | 27 |  |
| **Cancer stage – BCLC stage** | | | | | | | <0.001 |
| 0 | 2475 | 39.35 | 30.66 | 32 | 19 | 52 |  |
| A | 12300 | 33.37 | 29.97 | 26 | 13 | 44 |  |
| B | 7281 | 24.14 | 27.35 | 17 | 6 | 32 |  |
| C | 6386 | 21.66 | 26.16 | 14 | 3 | 29 |  |
| D | 448 | 25.33 | 33.54 | 13 | 1 | 34 |  |
| Unknown | 3894 | 25.54 | 30.51 | 16 | 5 | 34 |  |
| **Hospital level** | | | | | | | <0.001 |
| Medical centers | 20328 | 29.51 | 29.43 | 22 | 9 | 40 |  |
| Regional hospitals | 12319 | 26.70 | 29.28 | 19 | 7 | 35 |  |
| District hospitals | 137 | 29.33 | 33.16 | 18 | 4 | 45 |  |
| **Hospital ownership** | | | | | | | <0.001 ^b^ |
| Public | 10447 | 30.52 | 29.48 | 23 | 10 | 41 |  |
| Non-public | 22337 | 27.49 | 29.34 | 19 | 7 | 37 |  |

BCLC, Barcelona Clinic Liver Cancer classification; MDT, multidisciplinary team; NTD: New Taiwan Dollar; SD: standard deviation.

^a^. Kruskal-Wallis H test.

^b^. Wilcoxon rank-sum test.

Supplementary Table 2 Comparison of the diagnosis to the first treatment time interval of liver cancer patients with different characteristics, whether they join multidisciplinary team care.

| **Variables** | DTI of Non-MDT (Day) | | | | | | DTI of MDT (Day) | | | | | | *p* value |
| --- | --- | --- | --- | --- | --- | --- | --- | --- | --- | --- | --- | --- | --- |
|  | N | Mean | SD | Median | Q1 | Q3 | N | Mean | SD | Median | Q1 | Q3 |  |
| **Total** | 21856 | 27.91 | 29.65 | 20 | 7 | 38 | 10928 | 29.55 | 28.93 | 22 | 10 | 40 | <0.001 |
| **Gender** | | | | | | | | | | | | |  |
| Female | 5955 | 31.03 | 31.33 | 22 | 10 | 41 | 2982 | 31.49 | 29.88 | 25 | 11 | 42 | 0.015 |
| Male | 15901 | 26.74 | 28.91 | 19 | 7 | 36 | 7946 | 28.82 | 28.53 | 21 | 9 | 39 | <0.001 |
| **Age at diagnosed (years)** | | | | | | | | | | | | |  |
| ≦44 | 1667 | 21.84 | 26.45 | 14 | 4 | 30 | 842 | 22.90 | 24.60 | 17 | 6 | 32 | 0.027 |
| 45-54 | 4111 | 25.51 | 28.39 | 18 | 6 | 34 | 2087 | 28.14 | 28.35 | 21 | 9 | 37 | <0.001 |
| 55-64 | 6902 | 29.03 | 30.24 | 21 | 8 | 39 | 3423 | 30.27 | 29.45 | 23 | 10 | 41 | <0.001 |
| 65-74 | 6045 | 29.11 | 29.90 | 21 | 8 | 39 | 2981 | 30.65 | 28.70 | 24 | 11 | 41 | <0.001 |
| ≧75 | 3131 | 29.50 | 30.48 | 22 | 8 | 40 | 1595 | 31.30 | 30.52 | 24 | 10 | 44 | 0.005 |
| **Monthly salary (NTD)** | | | | | | | | | | | | |  |
| ≦ 20,008 | 1699 | 26.29 | 30.49 | 17 | 5 | 35 | 816 | 27.62 | 29.15 | 20 | 7 | 39 | 0.030 |
| 20,009-22,800 | 8318 | 28.24 | 30.01 | 20 | 7 | 38 | 4229 | 29.76 | 28.88 | 22 | 10 | 41 | <0.001 |
| 22,801-28,800 | 4789 | 29.21 | 30.62 | 20 | 8 | 40 | 2327 | 29.16 | 29.36 | 22 | 8 | 40 | 0.279 |
| 28,801-36,300 | 1930 | 27.04 | 28.27 | 19 | 8 | 36 | 934 | 30.95 | 30.30 | 22 | 10 | 42 | <0.001 |
| 36,301-45,800 | 2625 | 27.37 | 28.22 | 20 | 8 | 36 | 1356 | 29.44 | 27.96 | 22 | 10 | 41 | 0.002 |
| ≧ 45,801 | 2495 | 26.64 | 28.34 | 19 | 7 | 35 | 1266 | 29.87 | 28.10 | 24 | 11 | 39 | <0.001 |
| **Urbanization level** | | | | | | | | | | | | |  |
| Level 1 | 5097 | 28.24 | 29.69 | 20 | 8 | 38 | 2671 | 30.52 | 28.73 | 24 | 11 | 41 | <0.001 |
| Level 2 | 6144 | 28.32 | 29.73 | 20 | 8 | 38 | 3164 | 29.01 | 29.14 | 22 | 8.5 | 40 | 0.049 |
| Level 3 | 3679 | 27.01 | 28.65 | 19 | 7 | 37 | 1547 | 29.31 | 28.06 | 23 | 9 | 41 | <0.001 |
| Level 4 | 3642 | 27.06 | 29.41 | 19 | 7 | 36 | 1950 | 29.26 | 29.51 | 21 | 9 | 40 | <0.001 |
| Level 5-7 | 3294 | 28.55 | 30.76 | 20 | 7 | 38 | 1596 | 29.56 | 28.94 | 22 | 10 | 40 | 0.002 |
| **Charlson Comorbidity Index** | | | | | | | | | | | | |  |
| 0 | 1337 | 19.62 | 22.40 | 14 | 4 | 27 | 715 | 24.37 | 24.25 | 19 | 7 | 34 | <0.001 |
| 1 | 4917 | 25.68 | 27.60 | 18 | 7 | 34 | 2499 | 28.36 | 27.64 | 21 | 9 | 39 | <0.001 |
| 2 | 3562 | 28.67 | 29.52 | 21 | 9 | 38 | 1776 | 28.90 | 27.90 | 22.5 | 10 | 39 | 0.113 |
| ≧ 3 | 12040 | 29.51 | 30.97 | 21 | 8 | 40 | 5938 | 30.86 | 30.16 | 23 | 10 | 42 | <0.001 |
| **Severity of cirrhosis** | | | | | | | | | | | | |  |
| No cirrhosis | 12415 | 25.22 | 26.67 | 18 | 7 | 34 | 6311 | 27.34 | 26.56 | 21 | 9 | 37 | <0.001 |
| Mild cirrhosis | 8725 | 30.93 | 32.62 | 22 | 8 | 42 | 4250 | 32.30 | 31.30 | 25 | 10 | 44 | <0.001 |
| Severe cirrhosis | 716 | 37.64 | 34.70 | 29 | 13 | 52 | 367 | 35.60 | 35.25 | 26 | 12 | 50 | 0.236 |
| **History of anti-virus therapy** | | | | | | | | | | | | |  |
| No | 11334 | 26.68 | 29.30 | 19 | 6 | 36 | 5592 | 28.39 | 28.53 | 21 | 9 | 39 | <0.001 |
| Yes | 10522 | 29.22 | 29.96 | 21 | 9 | 39 | 5336 | 30.76 | 29.29 | 24 | 10 | 42 | <0.001 |
| **Tumor size (centimeters)** | | | | | | | | | | | | | |
| < 3 | 8678 | 35.13 | 31.70 | 27 | 14 | 46 | 4244 | 37.38 | 31.57 | 30 | 16 | 49 | <0.001 |
| 3-5 | 5245 | 28.92 | 29.37 | 21 | 9 | 38 | 2688 | 30.52 | 27.51 | 24.5 | 12 | 41.5 | <0.001 |
| > 5 | 7933 | 19.34 | 24.93 | 12 | 2 | 26 | 3996 | 20.57 | 23.98 | 14 | 4 | 28 | <0.001 |
| **Cancer stage – BCLC stage** | | | | | | | | | | | | |  |
| 0 | 1660 | 38.38 | 30.79 | 31 | 18 | 50 | 815 | 41.34 | 30.30 | 34 | 20 | 55 | 0.002 |
| A | 8227 | 32.73 | 30.31 | 25 | 12 | 43 | 4073 | 34.66 | 29.23 | 28 | 15 | 46 | <0.001 |
| B | 4817 | 23.41 | 27.19 | 16 | 5 | 31 | 2464 | 25.57 | 27.62 | 18 | 7 | 34 | <0.001 |
| C | 4260 | 21.20 | 26.55 | 13 | 3 | 28 | 2126 | 22.59 | 25.34 | 16 | 5 | 30 | <0.001 |
| D | 288 | 24.49 | 33.55 | 12 | 1 | 32.5 | 160 | 26.85 | 33.58 | 14 | 1 | 39 | 0.303 |
| Unknown | 2604 | 25.64 | 31.08 | 16 | 5 | 33 | 1290 | 25.36 | 29.34 | 16 | 5 | 35 | 0.638 |
| **Hospital level** | | | | | | | | | | | | | |
| Medical centers | 14275 | 28.50 | 29.84 | 20 | 8 | 38 | 6053 | 31.88 | 28.31 | 26 | 13 | 43 | <0.001 |
| Regional hospitals | 7478 | 26.75 | 29.16 | 19 | 7 | 35 | 4841 | 26.63 | 29.47 | 18 | 7 | 35 | 0.489 |
| District hospitals | 103 | 29.00 | 35.87 | 15 | 3 | 45 | 34 | 30.32 | 23.49 | 26 | 14 | 46 | 0.177 |
| **Hospital ownership** | | | | | | | | | | | | | |
| Public | 5976 | 29.16 | 30.04 | 21 | 8 | 40 | 4471 | 32.33 | 28.61 | 26 | 13 | 43 | <0.001 |
| Non-public | 15880 | 27.43 | 29.49 | 19 | 7 | 37 | 6457 | 27.62 | 28.99 | 20 | 8 | 36 | 0.292 |

BCLC, Barcelona Clinic Liver Cancer classification; DTI, diagnosis to first treatment time interval; MDT, multidisciplinary team; NTD, New Taiwan Dollar; SD, standard deviation.

Wilcoxon rank-sum test.

Supplementary Table 3 Bivariate analysis of survival outcomes for HCC patients based on various factors.

| **Variables** | Non-MDT | | | | | | MDT | | | | | | *p* value |
| --- | --- | --- | --- | --- | --- | --- | --- | --- | --- | --- | --- | --- | --- |
|  | Total | | Survival | | Death | | Total | | Survival | | Death | |  |
|  | N | % | n1 | % | n2 | ％ | N | % | n1 | % | n2 | % |  |
| Total | 21856 | 66.67 | 10653 | 48.74 | 11203 | 51.26 | 10928 | 33.33 | 5024 | 45.97 | 5904 | 54.03 | 0.206 |
| **Gender** | | | | | | | | | | | | | |
| Female | 5955 | 27.25 | 3093 | 51.94 | 2862 | 48.06 | 2982 | 27.29 | 1454 | 48.76 | 1528 | 51.24 | 0.403 |
| Male | 15901 | 72.75 | 7560 | 47.54 | 8341 | 52.46 | 7946 | 72.71 | 3570 | 44.93 | 4376 | 55.07 | 0.372 |
| **Age at diagnosed (years)** | | | | | | | | | | | | | |
| ≦ 44 | 1667 | 7.63 | 782 | 46.91 | 885 | 53.09 | 842 | 7.70 | 348 | 41.33 | 494 | 58.67 | 0.271 |
| 45-54 | 4111 | 18.81 | 2043 | 49.70 | 2068 | 50.30 | 2087 | 19.10 | 942 | 45.14 | 1145 | 54.86 | 0.033 |
| 55-64 | 6902 | 31.58 | 3632 | 52.62 | 3270 | 47.38 | 3423 | 31.32 | 1706 | 49.84 | 1717 | 50.16 | 0.344 |
| 65-74 | 6045 | 27.66 | 2861 | 47.33 | 3184 | 52.67 | 2981 | 27.28 | 1380 | 46.29 | 1601 | 53.71 | 0.210 |
| ≧ 75 | 3131 | 14.33 | 1335 | 42.64 | 1796 | 57.36 | 1595 | 14.60 | 648 | 40.63 | 947 | 59.37 | 0.527 |
| **Monthly salary (NTD)** | | | | | | | | | | | | | |
| ≦ 20,008 | 1699 | 7.77 | 714 | 42.02 | 985 | 57.98 | 816 | 7.47 | 299 | 36.64 | 517 | 63.36 | 0.756 |
| 20,009-22,800 | 8318 | 38.06 | 3879 | 46.63 | 4439 | 53.37 | 4229 | 38.70 | 1810 | 42.80 | 2419 | 57.20 | 0.216 |
| 22,801-28,800 | 4789 | 21.91 | 2255 | 47.09 | 2534 | 52.91 | 2327 | 21.29 | 1068 | 45.90 | 1259 | 54.10 | 0.657 |
| 28,801-36,300 | 1930 | 8.83 | 977 | 50.62 | 953 | 49.38 | 934 | 8.55 | 448 | 47.97 | 486 | 52.03 | 0.622 |
| 36,301-45,800 | 2625 | 12.01 | 1424 | 54.25 | 1201 | 45.75 | 1356 | 12.41 | 689 | 50.81 | 667 | 49.19 | 0.185 |
| ≧ 45,801 | 2495 | 11.42 | 1404 | 56.27 | 1091 | 43.73 | 1266 | 11.58 | 710 | 56.08 | 556 | 43.92 | 0.864 |
| **Urbanization level** | | | | | | | | | | | | | |
| Level 1 | 5097 | 23.32 | 2582 | 50.66 | 2515 | 49.34 | 2671 | 24.44 | 1269 | 47.51 | 1402 | 52.49 | 0.072 |
| Level 2 | 6144 | 28.11 | 3050 | 49.64 | 3094 | 50.36 | 3164 | 28.95 | 1511 | 47.76 | 1653 | 52.24 | 0.905 |
| Level 3 | 3679 | 16.83 | 1748 | 47.51 | 1931 | 52.49 | 1547 | 14.16 | 749 | 48.42 | 798 | 51.58 | 0.034 |
| Level 4 | 3642 | 16.66 | 1690 | 46.40 | 1952 | 53.60 | 1950 | 17.84 | 860 | 44.10 | 1090 | 55.90 | 0.975 |
| Level 5-7 | 3294 | 15.07 | 1583 | 48.06 | 1711 | 51.94 | 1596 | 14.60 | 635 | 39.79 | 961 | 60.21 | 0.005 |
| **Charlson Comorbidity Index** | | | | | | | | | | | | | |
| 0 | 1337 | 6.12 | 858 | 64.17 | 479 | 35.83 | 715 | 6.54 | 475 | 66.43 | 240 | 33.57 | 0.160 |
| 1 | 4917 | 22.50 | 2969 | 60.38 | 1948 | 39.62 | 2499 | 22.87 | 1525 | 61.02 | 974 | 38.98 | 0.122 |
| 2 | 3562 | 16.30 | 2184 | 61.31 | 1378 | 38.69 | 1776 | 16.25 | 1002 | 56.42 | 774 | 43.58 | 0.006 |
| ≧ 3 | 12040 | 55.09 | 4642 | 38.55 | 7398 | 61.45 | 5938 | 54.34 | 2022 | 34.05 | 3916 | 65.95 | 0.149 |
| **Severity of cirrhosis** | | | | | | | | | | | | | |
| No cirrhosis | 12415 | 56.80 | 6097 | 49.11 | 6318 | 50.89 | 6311 | 57.75 | 2986 | 47.31 | 3325 | 52.69 | 0.781 |
| Mild cirrhosis | 8725 | 39.92 | 4220 | 48.37 | 4505 | 51.63 | 4250 | 38.89 | 1893 | 44.54 | 2357 | 55.46 | 0.042 |
| Severe cirrhosis | 716 | 3.28 | 336 | 46.93 | 380 | 53.07 | 367 | 3.36 | 145 | 39.51 | 222 | 60.49 | 0.243 |
| **History of anti-virus therapy** | | | | | | | | | | | | | |
| No | 11334 | 51.86 | 4705 | 41.51 | 6629 | 58.49 | 5592 | 51.17 | 2291 | 40.97 | 3301 | 59.03 | 0.016 |
| Yes | 10522 | 48.14 | 5948 | 56.53 | 4574 | 43.47 | 5336 | 48.83 | 2733 | 51.22 | 2603 | 48.78 | <0.001 |
| **Tumor size (centimeters)** | | | | | | | | | | | | | |
| < 3 | 8678 | 39.71 | 5636 | 64.95 | 3042 | 35.05 | 4244 | 38.84 | 2631 | 61.99 | 1613 | 38.01 | 0.019 |
| 3-5 | 5245 | 24.00 | 2806 | 53.50 | 2439 | 46.50 | 2688 | 24.60 | 1354 | 50.37 | 1334 | 49.63 | 0.201 |
| > 5 | 7933 | 36.30 | 2211 | 27.87 | 5722 | 72.13 | 3996 | 36.57 | 1039 | 26.00 | 2957 | 74.00 | 0.002 |
| **Cancer stage – BCLC stage** | | | | | | | | | | | | | |
| 0 | 1660 | 7.60 | 1362 | 82.05 | 298 | 17.95 | 815 | 7.46 | 670 | 82.21 | 145 | 17.79 | 0.712 |
| A | 8227 | 37.64 | 5401 | 65.65 | 2826 | 34.35 | 4073 | 37.27 | 2598 | 63.79 | 1475 | 36.21 | 0.326 |
| B | 4817 | 22.04 | 1920 | 39.86 | 2897 | 60.14 | 2464 | 22.55 | 961 | 39.00 | 1503 | 61.00 | 0.029 |
| C | 4260 | 19.49 | 822 | 19.30 | 3438 | 80.70 | 2126 | 19.45 | 374 | 17.59 | 1752 | 82.41 | 0.002 |
| D | 288 | 1.32 | 68 | 23.61 | 220 | 76.39 | 160 | 1.46 | 33 | 20.63 | 127 | 79.38 | 0.183 |
| Unknown | 2604 | 11.91 | 1080 | 41.47 | 1524 | 58.53 | 1290 | 11.80 | 388 | 30.08 | 902 | 69.92 | <0.001 |
| **Treatment** | | | | | | | | | | | | | |
| Surgery | 5932 | 27.14 | 4381 | 73.85 | 1551 | 26.15 | 3265 | 29.88 | 2311 | 70.78 | 954 | 29.22 | 0.001 |
| Embolization | 1597 | 7.31 | 529 | 33.12 | 1068 | 66.88 | 499 | 4.57 | 148 | 29.66 | 351 | 70.34 | 0.606 |
| Radiotherapy | 891 | 4.08 | 124 | 13.92 | 767 | 86.08 | 443 | 4.05 | 73 | 16.48 | 370 | 83.52 | <0.001 |
| Surgery + local treatment | 3898 | 17.83 | 2640 | 67.73 | 1258 | 32.27 | 1866 | 17.08 | 1167 | 62.54 | 699 | 37.46 | 0.029 |
| Surgery + embolization | 1734 | 7.93 | 659 | 38.00 | 1075 | 62.00 | 348 | 3.18 | 119 | 34.20 | 229 | 65.80 | 0.554 |
| Embolization + chemotherapy | 3434 | 15.71 | 997 | 29.03 | 2437 | 70.97 | 1980 | 18.12 | 499 | 25.20 | 1481 | 74.80 | 0.327 |
| Embolization + radiotherapy + chemotherapy | 543 | 2.48 | 73 | 13.44 | 470 | 86.56 | 354 | 3.24 | 58 | 16.38 | 296 | 83.62 | 0.039 |
| Surgery + embolization + chemotherapy | 921 | 4.21 | 350 | 38.00 | 571 | 62.00 | 455 | 4.16 | 146 | 32.09 | 309 | 67.91 | 0.031 |
| Surgery + Local treatment +  embolization | 762 | 3.49 | 357 | 46.85 | 405 | 53.15 | 317 | 2.90 | 156 | 49.21 | 161 | 50.79 | 0.422 |
| Surgery + local treatment  + embolization + chemotherapy | 595 | 2.72 | 275 | 46.22 | 320 | 53.78 | 441 | 4.04 | 184 | 41.72 | 257 | 58.28 | 0.626 |
| Others treatment combination | 1549 | 7.09 | 268 | 17.30 | 1281 | 82.70 | 960 | 8.78 | 163 | 16.98 | 797 | 83.02 | 0.004 |
| **Hospital level** | | | | | | | | | | | | | |
| Medical centers | 14275 | 65.31 | 7065 | 49.49 | 7210 | 50.51 | 6053 | 55.39 | 3134 | 51.78 | 2919 | 48.22 | <0.001 |
| Regional hospitals | 7478 | 34.21 | 3537 | 47.30 | 3941 | 52.70 | 4841 | 44.30 | 1874 | 38.71 | 2967 | 61.29 | <0.001 |
| District hospitals | 103 | 0.47 | 51 | 49.51 | 52 | 50.49 | 34 | 0.31 | 16 | 47.06 | 18 | 52.94 | 0.749 |
| **Hospital ownership** | | | | | | | | | | | | | |
| Public | 5976 | 27.34 | 2936 | 49.13 | 3040 | 50.87 | 4471 | 40.91 | 2297 | 51.38 | 2174 | 48.62 | 0.002 |
| Non-public | 15880 | 72.66 | 7717 | 48.60 | 8163 | 51.40 | 6457 | 59.09 | 2727 | 42.23 | 3730 | 57.77 | <0.001 |

BCLC, Barcelona Clinic Liver Cancer classification; MDT, multidisciplinary team; NTD: New Taiwan Dollar.

Log-rank test analysis.

Supplementary Figure 1 Survival curves of patients with HCC according to BCLC stage.


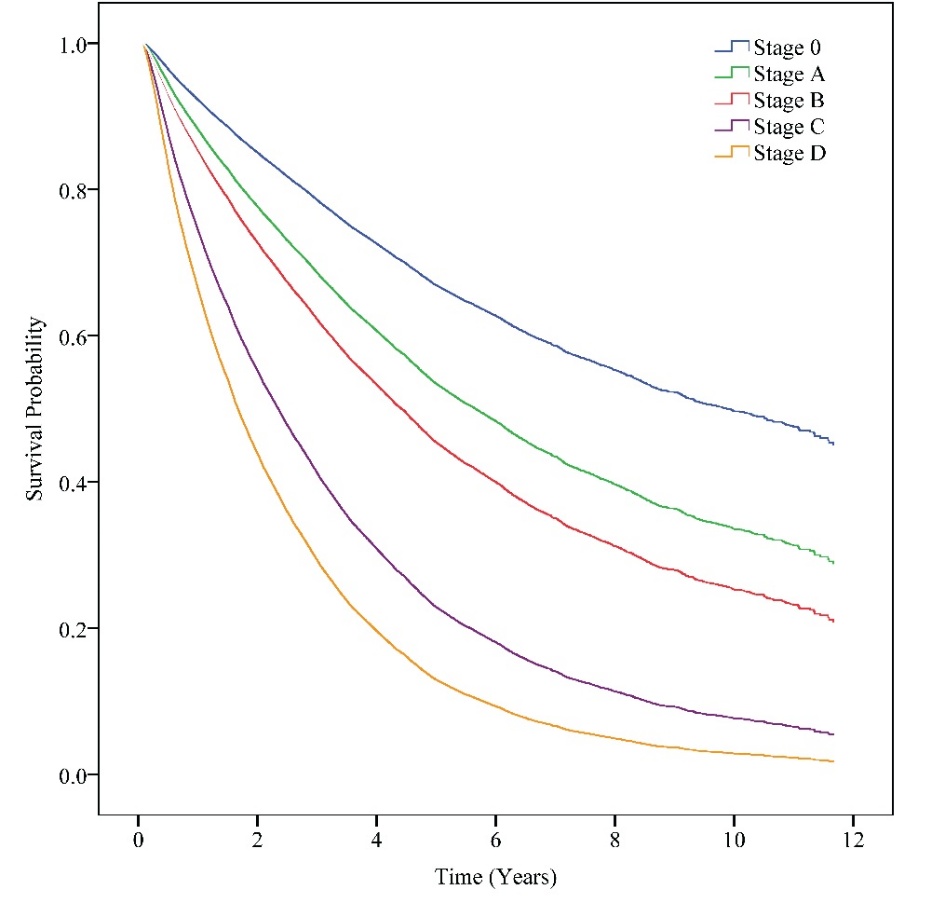


|  | HR | *p* value |
| --- | --- | --- |
| **BCLC stage** |  |  |
| Stage 0 | 1.00 |  |
| Stage A | 2.17 | <0.001 |
| Stage B | 4.14 | <0.001 |
| Stage C | 10.52 | <0.001 |
| Stage D | 42.12 | <0.001 |
